# CT and MRI radiomic features of lung cancer (NSCLC): comparison and software consistency

**DOI:** 10.1186/s41747-024-00468-8

**Published:** 2024-06-17

**Authors:** Chandra Bortolotto, Alessandra Pinto, Francesca Brero, Gaia Messana, Raffaella Fiamma Cabini, Ian Postuma, Agnese Robustelli Test, Giulia Maria Stella, Giulia Galli, Manuel Mariani, Silvia Figini, Alessandro Lascialfari, Andrea Riccardo Filippi, Olivia Maria Bottinelli, Lorenzo Preda

**Affiliations:** 1 Radiology Institute, Fondazione IRCCS Policlinico San Matteo, Pavia, 27100 Italy; 2 Diagnostic Imaging and Radiotherapy Unit, Department of Clinical, Surgical, Diagnostic, and Pediatric Sciences, University of Pavia, Pavia, 27100 Italy; 3 Department of Physics, University of Pavia, Via Bassi 6, Pavia, 27100 Italy; 4 Istituto Nazionale Di Fisica Nucleare, Sezione Di Pavia, Pavia, 27100 Italy; 5 Department of Mathematics, University of Pavia, Via Ferrata 5, Pavia, 27100 Italy; 6 Department of Medical Sciences and Infective Diseases, Unit of Respiratory Diseases, Fondazione IRCCS Policlinico San Matteo, Pavia, 27100 Italy; 7 Department of Internal Medicine and Medical Therapeutics, University of Pavia, Pavia, 27100 Italy; 8 Department of Political and Social Sciences, University of Pavia, Pavia, 27100 Italy; 9 Department of Radiation Oncology, Fondazione IRCCS Policlinico San Matteo, Pavia, 27100 Italy

**Keywords:** Biomarkers, Lung neoplasms, Magnetic resonance imaging, Radiomics, Tomography (x-ray computed)

## Abstract

**Background:**

Radiomics is a quantitative approach that allows the extraction of mineable data from medical images. Despite the growing clinical interest, radiomics studies are affected by variability stemming from analysis choices. We aimed to investigate the agreement between two open-source radiomics software for both contrast-enhanced computed tomography (CT) and contrast-enhanced magnetic resonance imaging (MRI) of lung cancers and to preliminarily evaluate the existence of radiomic features stable for both techniques.

**Methods:**

Contrast-enhanced CT and MRI images of 35 patients affected with non-small cell lung cancer (NSCLC) were manually segmented and preprocessed using three different methods. Sixty-six Image Biomarker Standardisation Initiative-compliant features common to the considered platforms, PyRadiomics and LIFEx, were extracted. The correlation among features with the same mathematical definition was analyzed by comparing PyRadiomics and LIFEx (at fixed imaging technique), and MRI with CT results (for the same software).

**Results:**

When assessing the agreement between LIFEx and PyRadiomics across the considered resampling, the maximum statistically significant correlations were observed to be 94% for CT features and 95% for MRI ones. When examining the correlation between features extracted from contrast-enhanced CT and MRI using the same software, higher significant correspondences were identified in 11% of features for both software.

**Conclusions:**

Considering NSCLC, (i) for both imaging techniques, LIFEx and PyRadiomics agreed on average for 90% of features, with MRI being more affected by resampling and (ii) CT and MRI contained mostly non-redundant information, but there are shape features and, more importantly, texture features that can be singled out by both techniques.

**Relevance statement:**

Identifying and selecting features that are stable cross-modalities may be one of the strategies to pave the way for radiomics clinical translation.

**Key points:**

• More than 90% of LIFEx and PyRadiomics features contain the same information.

• Ten percent of features (shape, texture) are stable among contrast-enhanced CT and MRI.

• Software compliance and cross-modalities stability features are impacted by the resampling method.

**Graphical Abstract:**

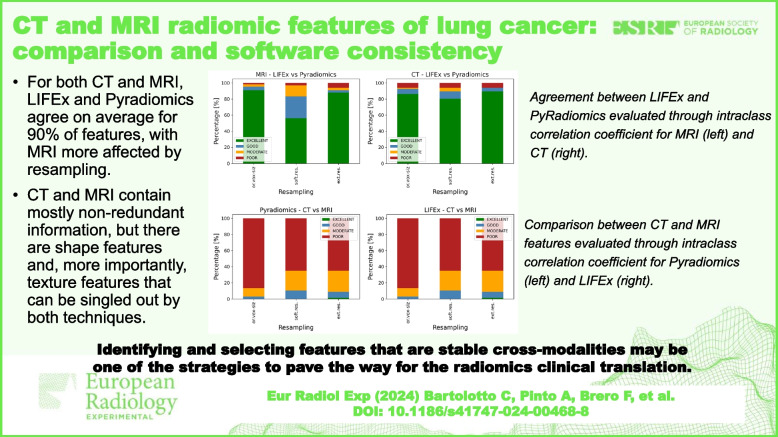

## Background

Radiomics is a quantitative method that allows extracting mineable high-dimensional data, named radiomic features, from digital medical images [1–3]. The core hypothesis is that radiomic features might integrate visual analysis by unveiling tissue details and heterogeneity linked to image intensity distribution at an almost microscopic scale [4–6], thus becoming novel biomarkers. In oncology, radiomic features could, in principle, describe both the microenvironment [7] and the histotype and genotype of the tumor mass, supporting diagnosis determination, defining the prognosis, and predicting the therapeutic response [8]. The widespread interest in this method arises from several aspects. Radiomics, unlike biopsy, non-invasively captures information about the entire tumor [9]. Moreover, radiomics allows several evaluations of diagnostic images that are routinely acquired and permits to conduct analyses at different time points [10]. Radiomic features can be divided into four main categories: shape, histogram-based, texture, and wavelet [11]. In general, shape ones refer to the geometric properties of a region of interest; first-order features are related to the image intensity histograms; texture features define the mathematical relationship of a single voxel with one or more neighboring voxels reflecting, *e.g.*, the intratumoral heterogeneity; and wavelet features are filter-based features able to enhance some characteristics of the image, analyzing its frequency domain information [12, 13].

Over the years, lung cancer has been the subject of several radiomic studies, being the second most frequent cancer and the leading cause of cancer-related death worldwide [14]. Many studies have been conducted by using computed tomography (CT) and positron emission tomography (PET) acquisitions, which are already widely used in the daily management of lung cancer [15–19]. Despite the great interest in integrating lung magnetic resonance imaging (MRI) into clinical practice, the lung remains one of the few anatomical sites in which MRI has not yet reached CT performances [20, 21]. Even though MRI does not expose patients to ionizing radiation and provides optimal soft tissue contrast and exclusive morpho-functional information, it is still underused due to unfavorable occurrences [22].

The main obstacles to obtain good lung MRI images are related to the low signal-to-noise ratio (SNR) caused by lung parenchyma’s poor proton density, frequent tissue-air interfaces, movement artifacts, and the lack of standard protocols [23]. In the literature, there are few studies concerning the extraction of radiomic features from MRI of lung tumors. For example, one study determined the optimal timing post-contrast injection to extract radiomic features on T1-weighted images for predicting 2 years of progression-free survival [24]. Another preliminary study suggested that MRI-derived radiomic features may improve the accuracy of models that predict therapy response and survival at different time points, compared to that of models based on CT features only [10]. Similarly, few studies have investigated the correlation between radiomic features extracted from different imaging techniques. Mahon et al. [10] have investigated the repeatability of texture features derived from CT and MRI of lung cancer. On the other hand, Vuong et al. [13] have analyzed the correlation between features extracted from PET/CT and PET/MRI images, finding a close correlation between them.

In this scenario, we aimed to conduct a preliminary methodological investigation, exploring the correlation between CT and MRI lung cancer radiomic features, to assess whether specific lung cancer intrinsic aspects can be depicted by both imaging modalities. Since CT and MRI are based on different physical principles, radiomic features extracted with these techniques are generally not directly comparable, even considering the lower-level ones. CT scans employ x-rays to produce detailed images of the body, which describe tissue’s electronic density, while MRI uses magnetic fields and radiofrequency pulses to generate images that reflect tissue’s complex properties such as proton density, nuclear relaxation times, and many other, including parameters related to functional behavior. Consequently, we hypothesize that if some features are directly comparable between CT and MRI, we could assume that they are strongly related to tissue/organ biology and physiology.

To evaluate the correlation between CT and MRI features, we employed two open-access software, LIFEx [25] and PyRadiomics [26], with a double purpose. The first objective was to evaluate the correlation between LIFEx and PyRadiomics features, chosen on a broad range, as reported also in literature [27, 28], for two different imaging modalities evaluated separately on the same patient cohort. The second objective was to establish possible correlations among CT and MRI radiomic features, determining at the same time which software enables the extraction of such features. Lastly, we evaluated the impact of the voxel resampling algorithm on the two previous goals, as is known from the literature that the choice made at this stage can impact the analysis [29, 30].

## Methods

### Patients

This study was approved by the local Medical Research Ethics Committee of Fondazione IRCSS Policlinico San Matteo (Protocol code P_20130113422), and informed consent was obtained from all participants.

Thirty-five patients with non-small cell lung cancer (NSCLC), histologically confirmed from April 2021 to June 2023, were prospectively included as the study participants. The cohort consists of 26 males (74%) and 9 females (26%), with ages ranging from 49 to 84 years (median age 68 years). Concerning the NSCLC histotype distribution, 13 patients (37%) had an adenocarcinoma, 12 patients (34%) a squamocellular carcinoma, and the remaining 10 (29%) a poorly differentiated NSCLC. Tumor size was between 2 cm and a maximum of 15 cm with a corresponding stage between the II and the IV stage (in particular, 4 of II, 20 of III, and 11 of IV).

The following patients were excluded: (i) patients who had no adequate compliance capabilities and/or characteristics for undergoing MRI (*e.g.*, claustrophobia, contraindications to MRI such as pacemakers, contraindications to Gd-based contrast agents); (ii) those who had received treatment before imaging; (iii) patients with lung tumors not classified as NSCLC.

### Image acquisition

#### CT protocol

All patients underwent a thoracic CT examination, in a supine position from the apex to the base of the lung. Conventional CT was performed with a 64-slice scanner (SOMATOM Flash; Siemens Healthineers, Erlangen, Germany) for 16 patients, with a 16-slice scanner (SOMATOM Sensation; Siemens Healthineers) for 8 patients, with a 64-slice scanner (SOMATOM Sensation; Siemens Healthineers) for 6 patients, with a 160-slice scanner (Aquilion PRIME; Canon Medical Systems, Otawara, Japan) for 2 patients, and with a 320-slice CT (Aquilion ONE; Canon Medical Systems) for 3 patients. The scanning parameters were tube voltage 120 kV, tube current automatically modulated, slice thickness 2 mm, slice spacing 1 mm, pitch 1, rotation time 0.5 s, matrix 384 × 384, field of view set to 300 mm, and then adapted to patients. The scanning was completed under breath-hold condition and directly after the intravenous iodinated contrast medium injection (iomeprol 350 mgI/mL, 2 mL/s, 120 mL, 40-mL saline flush), in the venous phase (*i.e.*, 60−90 s after injection). After scanning, the original images were set to be the mediastinal window (smooth-medium kernel), automatically reconstructed.

#### MRI protocol

All patients underwent thoracic MRI in a supine position from the apex to the base of the lung. Conventional MRI was performed with a 1.5-T system (MAGNETOM Aera; Siemens Healthineers) using a 32-channel surface coil. During the examination, both free-breathing and breath-hold sequences were used. Scan sequences included in the study were axial and coronal volumetric interpolated breath-hold examination − VIBE T1-weighted sequences after the intravenous injection of paramagnetic contrast medium (gadoterate meglumine, 0.2 mL/kg (0.1 mmol/kg), 2 mL/s, 40-mL saline flush). For axial scanning, the matrix was 320 × 320, repetition time 2.1 ms, echo time 0.72 ms, field of view 450 × 350 mm, slice thickness 2.5 mm, layer spacing 0 mm, and number of layers 96.

### Image selection

Chest axial CT images and axial MRI T1-weighted images after contrast medium injection were selected for feature extraction in this study. We opted to include only contrast-enhanced T1-weighted MRI images to facilitate a direct comparison with the portal-venous phase CT scans. This choice was driven by the need to select the MRI sequence that best resembles the CT phase from a visual radiological and pharmacokinetic point of view.

### Tumor segmentation

Contrast-enhanced axial chest CT axial T1-weighted MRI in the Digital Imaging and Communications in Medicine − DICOM format were imported into the ITK-SNAP software (http://www.itksnap.org) and manually segmented.

The segmentation of CT images was made semiautomatically, using a Hounsfield unit seed-based method, while the segmentation of MRI was performed completely manually considering the lack of automatic or semiautomatic options. The segmentations were performed by three different radiologists (A.P., G.M., and C.B. with 2, 4, and 7 years of experience in thoracic imaging); complex cases were reviewed collegially with the aid of a fourth expert thoracic radiologist (L.P.). Cases were randomly assigned to each operator, and CT and MRI were not presented simultaneously; the minimal interval period between CT and MRI segmentation was 21 days. The region of interest of the tumor was segmented slice by slice to obtain the whole volume of interest by summing the segmented areas on each slice; the original images and the corresponding volume of interest image were saved using the Neuroimaging Informatics Technology Initiative − NIfTI-1format (https://nifti.nimh.nih.gov/nifti-1).

### Image preprocessing

As suggested by previous literature [31–35] to reduce variability between images, it is necessary to preprocess the images. Regarding radiomic features, some image characteristics are more influent than others. In particular, the gray-level distribution and voxel size are highly relevant. Regarding the voxel resampling, three different strategies have been considered:

1. Features have been computed without performing resampling (original voxel size).
2. Features have been computed after setting resampling voxel dimensions directly on the software considered (software resampling).
3. All images have been resampled into the same voxel space using the Python package Nibabel (https://github.com/nipy/nibabel) before performing the radiomic features extraction (external resampling).

For the second and third resampling modalities, the voxel size has been set to 1 × 1 × 1 mm^3^ for CT and 1.4 × 1.4 × 1.4 mm^3^ for MRI.

We have also normalized the signal intensity distribution of MRI images through the histogram matching technique, as proposed in previous works [36]. Specifically, by using the SimpleITK Python library [37], we transformed the intensity histogram of the images to align with the histogram of a reference image from a healthy subject. As the final preprocess step, we have discretized the image gray level distribution in 64 bins.

### Feature selection and extraction

We used the freeware LIFEx [25] version 7.3.0 and the open-source Python package PyRadiomics [26] version 3.0.1, Image Biomarker Standardisation Initiative (IBSI) [31] compliant. Both platforms allow to customize several parameters (spatial resampling, rescaling, and so on). The spatial resampling customization was performed just for the internal method resampling. The interpolator chosen is the sitkBSpline [38] and the intensity range was discretized in 64 bins, extracting features fixing the bin number, as advised when intensity units are arbitrary, as is for MRI [31]. We selected 66 IBSI-compliant features common to both software reported in Table 1. The extracted features were from six different feature categories: first-order features, shape features, and features from four different textures subdomains: gray-level co-occurrence matrix (GLCM), gray-level run length matrix (GLRLM); gray-level size zone matrix (GLZLM); and neighboring gray-tone difference matrix (NGTDM) [8].

**Table 1:** List of all 66 IBSI-compliant features common to both software (LIFEx and PyRadiomics), divided into different classes

Shape	Histogram		GLCM		GLRLM	GLZLM	NGTDM
Voxel Volume Surface Area Sphericity Maximum3DDiameter	Skewness Kurtosis Entropy Energy Uniformity Mean Median Minimum	10th Percentile 90th Percentile Maximum InterquantileRange Range MeanAbsolute Deviation (MAD) RobustMean AbsoluteDeviation(rMAD) Variance	Contrast Correlation Dissimilarity Energy Entropy InverseDifference Autocorrelation JointAverage ClusterProminance ClusterTendency	ClusterShade DifferenceVariance DifferenceEntropy InverseVariance SumEntropy JointVariance JointMaximum NormalizedInverse Difference (NID)	Short Run Emphasis (SRE) Long Run Emphasis (LRE) Gray Level Non-Uniformity (GLNU) Run Length Non-Uniformity (RLNU) Run Percentage (RP) Low Gray Level Run Emphasis (LGRE) High Gray Level Run Emphasis (HGRE) Short Run Low Gray Level Emphasis (SRLGE) Short Run High Gray Level Emphasis (SRHGE) Long Run Low Gray Level Emphasis (LRLGE) Long Run High Gray Level Emphasis (LRHGE)	Small Zone Emphasis (SZE) Large Zone Emphasis (LZE) Gray Level Non Uniformity (GLNU) Zone Size Non Uniformity (ZLNU) Zone Percentage (ZP) Gray Level Variance (GLV) Low Gray Level Zone Emphasis (LGZE) High Gray Level Zone Emphasis (HGZE) Small Zone Low Gray Level Emphasis (SZLGE) Small Zone High Gray Level Emphasis (SZHGE) Large Zone Low Gray Level Emphasis (LZLGE) Large Zone High Gray Level Emphasis (LZHGE)	Coarseness Complexity Busyness Strength Contrast

*GLCM* Gray-level co-occurrence matrix*, GLRLM* Gray-level run length matrix*, GLZLM* Gray-level size zone matrix, *NGTDM* Neighboring gray-tone difference matrix

### Statistical analysis

All the statistical analysis has been conducted using Python (version 3.8.10, http://www.python.org). To assess the features’ agreement between the two radiomic software (for the same imaging modality) and between the two different imaging modalities (for the same software), the intraclass correlation coefficient (ICC) has been calculated (two-way mixed effects, absolute agreement single measurement configuration [39]). The ICC value was calculated as follows:

$${\text{ICC}}= \frac{{{\text{MS}}}_{{\text{R}}}- {{\text{MS}}}_{{\text{E}}}}{{{\text{MS}}}_{{\text{R}}}+\left(k-1\right){{\text{MS}}}_{{\text{E}}}+\frac{k}{n}({{\text{MS}}}_{{\text{C}}}-{{\text{MS}}}_{{\text{E}}})}$$

where *MS*_*R*_ = mean square for rows; *MS*_*E*_ = mean square for error; *MS*_*C*_ = mean square for columns; *n* = number of subjects; and *k* = number of raters/measurements. We divided the ICC values into four ranges: poor (ICC < 0.5), moderate (0.5 ≤ ICC < 0.75), good (0.75 ≤ ICC < 0.9), and excellent (ICC ≥ 0.9) reliability.

## Results

### Agreement between LIFEx and PyRadiomics software

**Fig. 1:**
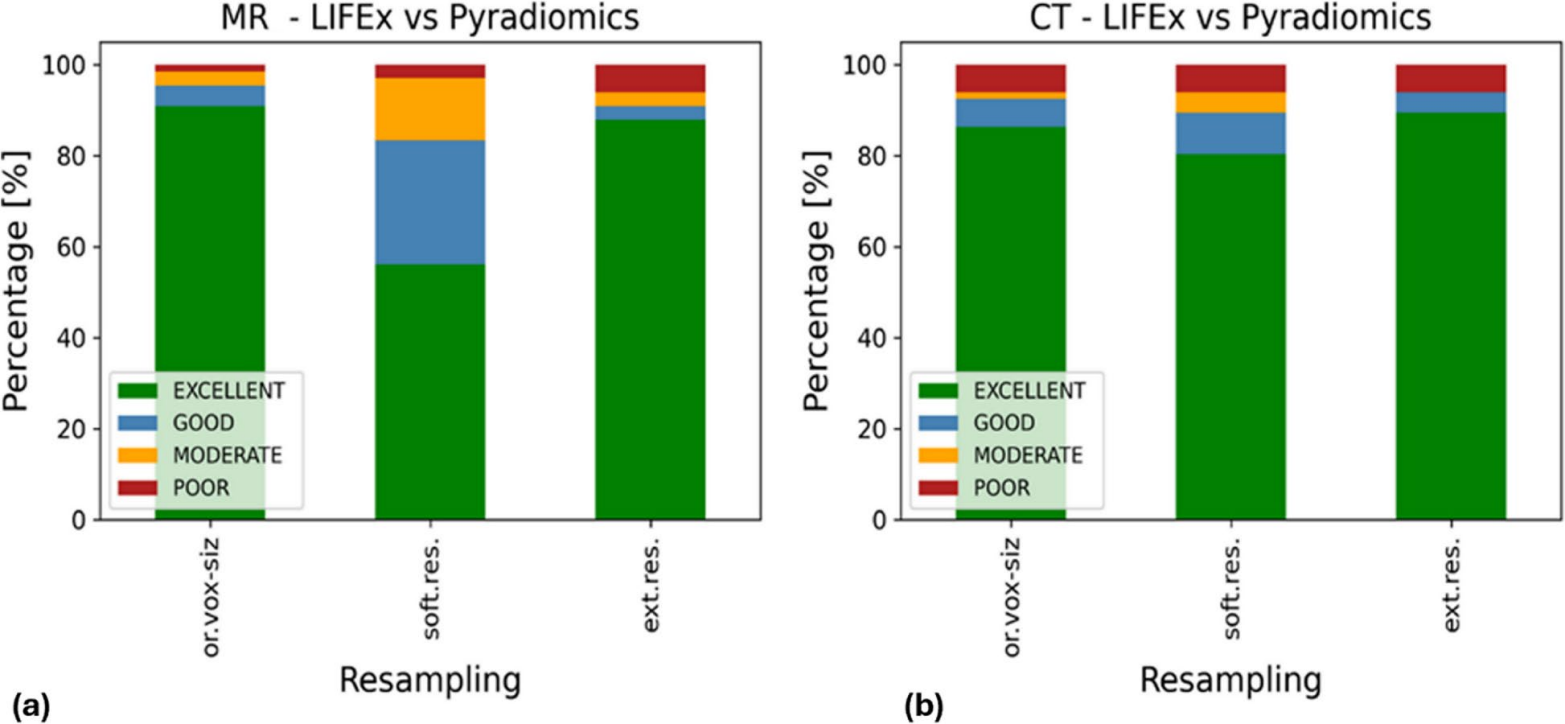
Consistency between radiomic features extracted with LIFEx and PyRadiomics, expressed through ICC, from MRI (**a**) and CT (**b**) images. *Ext.res.* External resampling, *Or.vox-siz.* Original voxel size, *Soft.res.* Software resampling

The agreement between features computed from LIFEx and PyRadiomics was assessed for both CT and MRI and the three different voxel resampling strategies. The ICC values divided into four confidence levels for MRI (left) and CT (right) are shown in Fig. 1.

As regards MRI, excellent or good reliability was achieved by 95% of features without voxel resampling, 83% of features with the internal resampling software, and 91% of features with the external resampling software, as summarized in Table 2. Poor or moderate reliability was observed for 5% of features without any voxel resampling, 17% of features with the internal resampling software, and 9% of features with the external resampling software. For each of the resampling methods, moderate reliability was associated with MAX3DDiameter (SHAPE) and Sum Entropy (GLCM). Moreover, the feature Inverse Variance (GLCM) exhibited poor reliability across all three resampling strategies.

**Table 2:** LIFEx *versus* PyRadiomics. Agreement between LIFEx and PyRadiomics for both MRI and CT, divided by ICC ranges. The results are presented in terms of radiomic features percentage exhibiting specific ICC values across the considered resampling methods

Intraclass correlation coefficient					
Imaging technique	Resampling	Excellent [%]	Good [%]	Moderate [%]	Poor [%]
MRI	Or.vox-siz	91.0	4.5	3.0	1.5
	Soft.res	56.1	27.3	13.6	3.0
	Ext.res	87.9	3.0	3.0	6.1
CT	Or.vox-siz	86.0	6.1	1.5	6.1
	Soft.res	80.3	9.1	4.5	6.1
	Ext.res	89.4	4.5	0	6.1

*Ext.res* External resampling, *Or.vox-siz.* Original voxel size, *Soft.res.* Software resampling

**Fig. 2:**
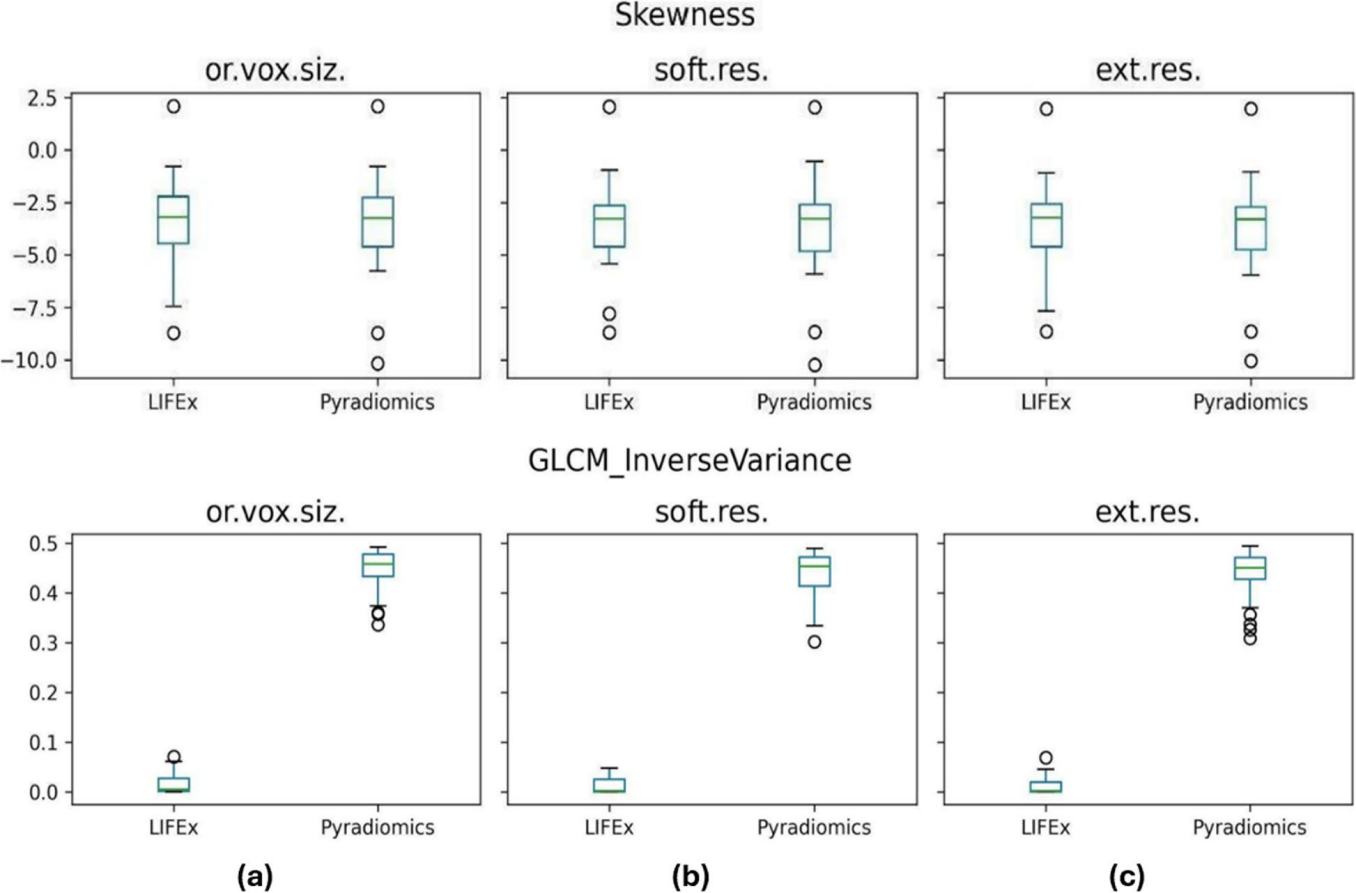
Comparison between features distributions computed by LIFEx and PyRadiomics platforms from MRI acquisitions. The top panel line represents a feature with excellent reliability (Skewness), while the bottom panel line a feature with poor reliability (GLCM Inverse Variance) for the three options considered: original voxel size (**a**); software (internal) resampling (**b**); external resampling (**c**). *Ext.res.* External resampling, *Or.vox-siz.* Original voxel size, *Soft.res.* Software resampling

**Fig. 3:**
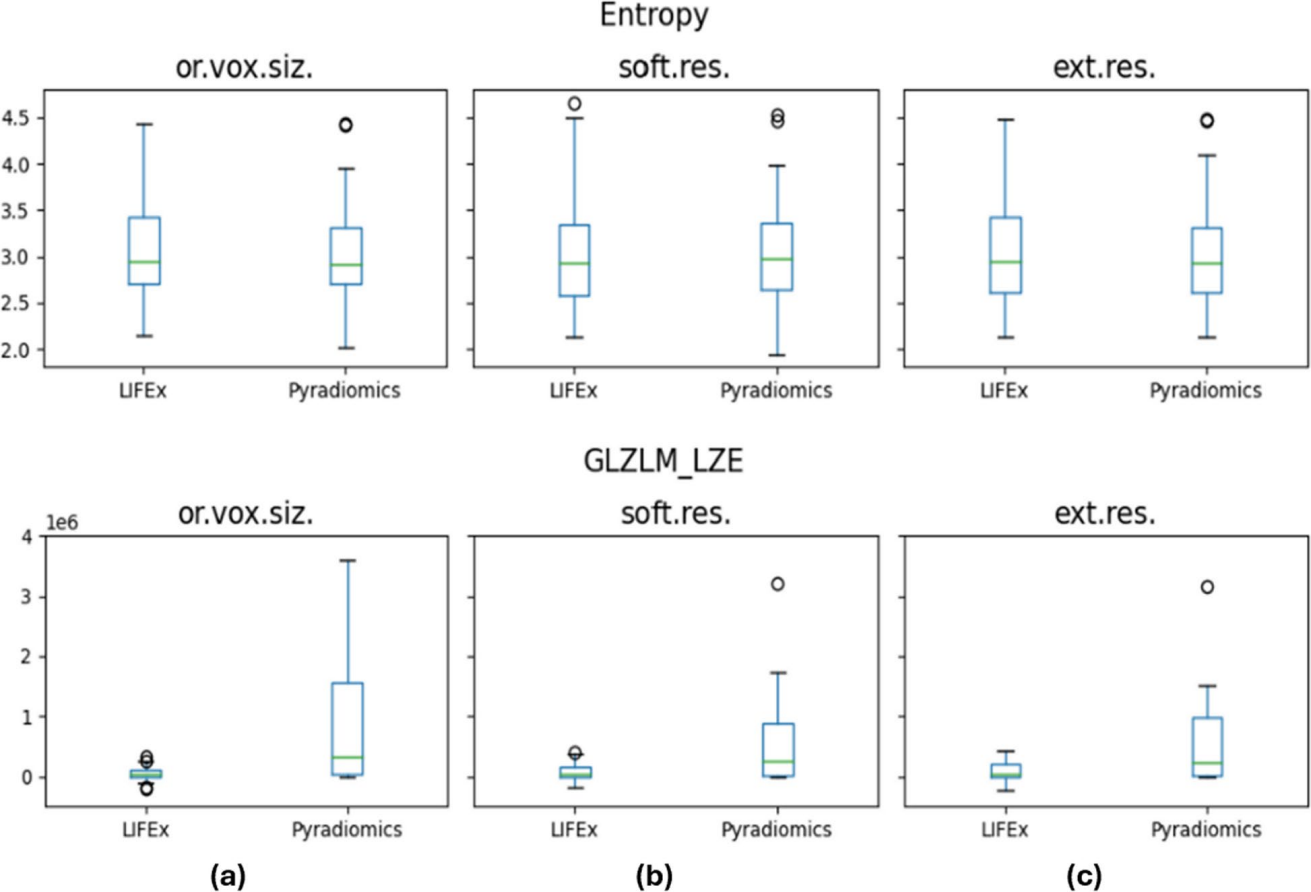
Comparison between feature distributions computed by LIFEx and PyRadiomics platforms from CT images. The top panel line shows a feature with excellent reliability (Entropy), while the bottom panel line displays a feature with poor reliability (GLZLM Large Zone Emphasis), for the three options considered: original voxel size (**a**); software (internal) resampling (**b**); external resampling (**c**). *Ext.res.* External resampling, *Or.vox-siz.* Original voxel size, *Soft.res.* Software resampling

Considering CT images, 92% of features demonstrated excellent or good reliability without voxel resampling, 89% of features had excellent or good reliability with internal resampling, and 94% of features exhibited excellent or good reliability with external resampling, as summarized in Table 2. Poor or moderate reliability was observed for 8% of features without voxel resampling, 11% of features with internal resampling, and 6% of features with external resampling. The three resampling strategies exhibited poor reliability for GLCM Inverse Variance, GLZLM LZE, GLZLM LZHGE, and GLZLM LZLGE. Figures 2 and 3 illustrate the variability of LIFEx-PyRadiomics features values for MRI and CT, respectively. These figures represent the distributions of a feature with excellent ICC and one with poor ICC.

### Correlation between CT and MRI features

**Fig. 4:**
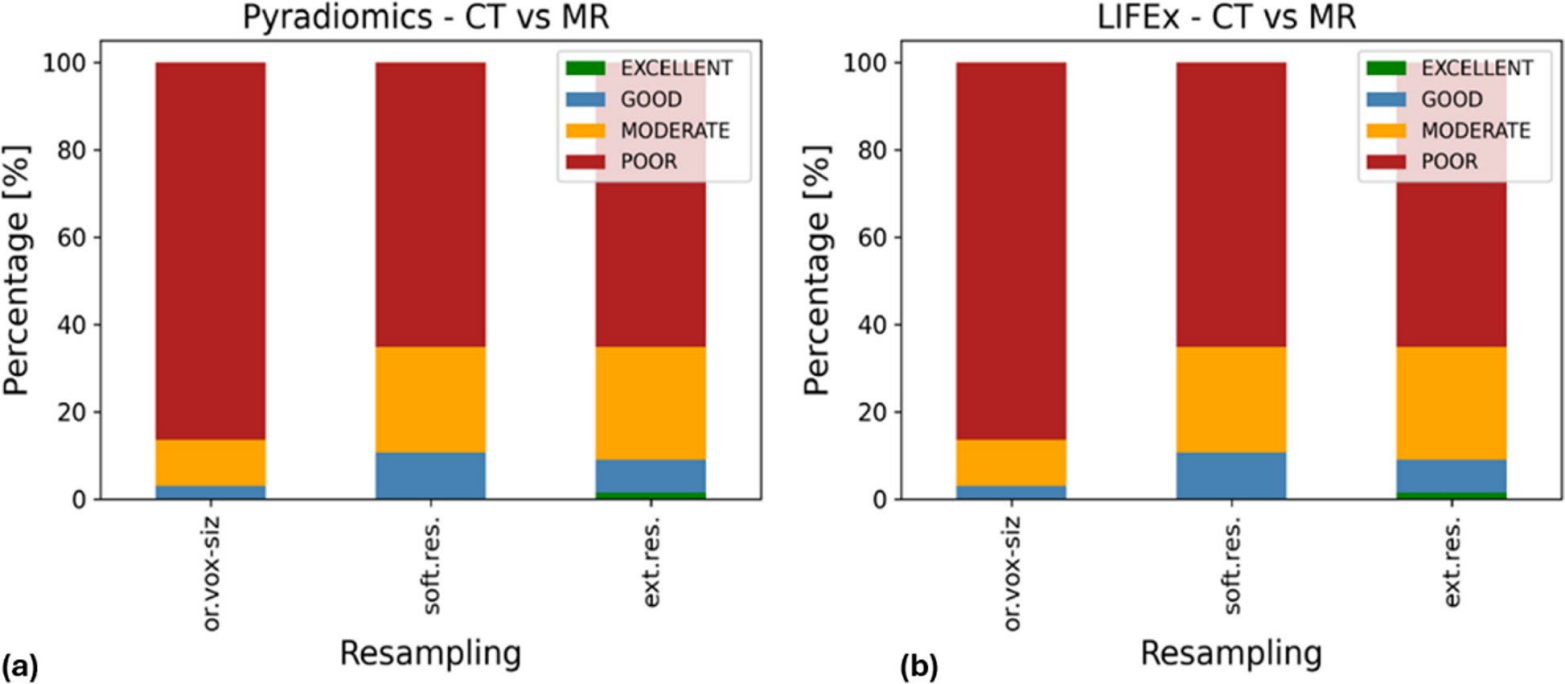
Agreement between radiomic features extracted from CT and MRI images, expressed through ICC, for PyRadiomics (**a**) and LIFEx (**b**). *Ext.res.* External resampling, *Or.vox-siz.* Original voxel size, *Soft.res.* Software resampling

The comparison between features computed from CT and MRI was performed for the three voxel resampling strategies and the two radiomic software separately. Figure 4 summarizes the distribution of the ICC values in the four ranges of agreement. As regards PyRadiomics, only 3% of features had excellent or good reliability without voxel resampling, 11% of features showed excellent or good reliability with internal resampling, and 9% of features possessed excellent or good reliability with external resampling (Table 3). A poor or moderate agreement was obtained for 97% of features without voxel resampling, 89% of features with internal resampling, and 91% of features with external resampling. Table 4 provides a summary of the features with excellent and good reliability. Excellent or good agreement was observed between features extracted from different imaging modalities for SHAPE features Volume and Surface Area, as well as for a few texture-based features. When considering all the analyzed resampling methods, the features demonstrating good/excellent reliability across all methods are the SHAPE ones. However, focusing on both internal and external resampling reveals additional common features, NGTDM Busyness, NGTDM Strength, and GLZLM ZP.

**Table 3:** CT *versus* MRI. CT-MRI concordance expressed through the percentage of radiomic features exhibiting a specific ICC, for both LIFEx and PyRadiomics. The results are reported for each of the considered resampling methods

Intraclass correlation coefficient					
Radiomic software	Resampling	Excellent [%]	Good [%]	Moderate [%]	Poor [%]
LIFEx	Or.vox-siz	0	4.5	7.5	88
	Soft.res	0	9.1	27.3	63.6
	Ext.res	1.5	9.1	22.7	66.7
PyRadiomics	Or.vox-siz	0	3.0	10.6	86.4
	Soft.res	0	10.6	24.2	65.2
	Ext.res	1.5	7.6	25.8	65.1

*Ext.res.* External resampling, *Or.vox-siz.* Original voxel size, *Soft.res.* Software resampling

**Table 4:** Radiomic features showing excellent and good ICC coefficients in CT-MRI comparison, considering PyRadiomics and LIFEx. For both software, the sole feature with excellent ICC is Voxel Volume (SHAPE), only employing external resampling

PyRadiomics software			LIFEx software		
Original voxel size	Internal resampling	External resampling	Original voxel size	Internal resampling	External resampling
SHAPE_Voxel Volume SHAPE_Surface Area	SHAPE_Voxel Volume SHAPE_Surface Area NGTDM_Busyness NGTDM_Strength GLZLM_ Zone_Percentage_(ZP) GLCM_DifferenceEntropy GLCM_NID	SHAPE_Voxel Volume SHAPE_Surface Area NGTDM_Busyness NGTDM_Strength GLZLM_Zone_Percentage(ZP) GLRLM_RLNU	SHAPE_Voxel Volume SHAPE_Surface Area SHAPE_Maximum3DDiameter	SHAPE_Voxel Volume SHAPE_Surface Area SHAPE_Maximum3DDiameter NGTDM_Busyness NGTDM_Strength GLRLM_RLNU	SHAPE_Voxel Volume SHAPE_Surface Area SHAPE_Maximum3DDiameter NGTDM_Busyness NGTDM_Strength GLRLM_RLNU GLZLM_ Zone_Percentage (ZP)

*GLCM* Gray-level co-occurrence matrix*, GLRLM* Gray-level run length matrix*, GLZLM* Gray-level size zone matrix, *NGTDM* Neighboring gray-tone difference matrix*, NID* Normalized inverse difference, *RLNU* Run length non-uniformity

Considering LIFEx, reliability between imaging modalities was excellent or good for 5% of features without voxel resampling, 9% of features with internal resampling, and 11% of features with external resampling (Table 3). Reliability was poor and moderate for 95% of features without voxel resampling, 91% of features with internal resampling, and 89% of features with external resampling. Features with good agreement between imaging modalities belong to SHAPE features (Volume, Surface Area, and MAX3D Diameter) and texture features, as detailed in Table 4. Only the SHAPE feature Volume resulted in an excellent ICC for the external resampling. As found for PyRadiomics, texture features presented good ICC just for internal and external resampling. In this case, features common to both resampling methods, with good ICC, are NGTDM Busyness and Strength, and GLRLM RLNU.

## Discussion

The first part of our study evaluated the correlation between features extracted by LIFEx and PyRadiomics from contrast-enhanced CT and MRI of NSCLCs. Our purpose is to verify whether there is an agreement between the two radiomic software, considering two different imaging techniques.

For what concerns MRI, at least 83% of the features showed a good or excellent ICC for each of the considered resampling methods. The maximum agreement between LIFEx and PyRadiomics was obtained for images with original voxel dimensions (94.5% of the features), while the minimum agreement was gained by the internal resampling method (83% of the features). Possibly, the result found in this last case arises from the distinct resampling algorithms implemented in LIFEx and PyRadiomics. This effect could have been emphasized also by the upsampling operation made on the original images. In particular, the in-plane dimension was conserved, while the *z*-axis has been modified to obtain an isotropic voxel.

Considering CT images, we have found that at least 89% of features showed a good/excellent ICC for each of the considered resampling methods. The highest agreement between LIFEx and PyRadiomics was obtained for external resampling. The percentage of CT features exhibiting good/excellent reliability showed less variation among the resampling methods, compared to MRI. This could be addressed by the fact that CT images exhibit lower noise levels when compared to MRI ones. Moreover, the original voxel dimensions of CT images were closer to the isotropic voxel size than those of MRI, potentially reducing the impact of resampling on the distribution of extracted features. CT features that revealed moderate/poor reliability are the same for each of the resampling methods: GLCM Inverse Variance, GLZLM LZE, GLZLM LZHGE, and GLZLM LZLGE.

Some considerations can be made for both CT and MRI. Firstly, the software agreement was higher when considering external resampling compared to internal resampling. This once again underlines the feature’s dependency on the choices made in each step of the radiomic workflow. Secondly, considering the high percentage of concordant features between LIFEx and PyRadiomics for both CT and MRI, it is possible to conclude that the two software agree with each other, regardless of the imaging technique used. These results were achieved following the IBSI guidelines and the conclusions obtained from previous works [27, 31]. It is noteworthy that LIFEx-PyRadiomics agreement was achieved considering two different imaging techniques. This is an important result, especially for lung MRI, given the challenges posed by artifacts, low signal, and other well-known limits.

The second part of the study investigated which of the two software packages could extract the highest number of correlated features among CT and MRI. Our purpose was to verify whether there is some lung cancer intrinsic information that can be depicted by both CT and MRI. We were not expecting a high number of features to be correlated between the two imaging modalities, as they rely on different physical principles, even though the local effect of iodine and Gd-based agents is an increase in x-ray attenuation (CT scans) or signal (T1-weighted MRI), which always translates in an increase of the values of the gray scale in the images. We aimed to address the question regarding the possible existence of information strictly linked to NSCLC biology, beyond the considered imaging technique.

From the medical point of view, it should be noticed that the low percentage (10%) of correlated radiomic features between MRI and CT can be considered informative about lung cancer. Features stable across modalities may carry relevant biological information, showing the ability to reflect histopathological phenomena, such as inflammation or vascularization, related to lung cancer’s characterization and options for treatment. Thus, with higher statistics of patients, selecting features that are cross-modality and stable (even few) may be one of the strategies to pave the way for the clinical translation of radiomic biomarkers.

Regardless of the three resampling methods, most of the features showed an ICC < 0.5, *i.e.*, a very low number of features with excellent and good agreement was extracted by a single software from CT and MRI (see Table 3). In this case, the percentage of CT-MRI features highly correlated for both the analyzed software was higher considering resampled images rather than the original ones. The features with good ICC, regardless of the resampling method, are the SHAPE features Volume and Surface Area for both software, plus Maximum3DDiameter SHAPE for LIFEx. Moreover, for both PyRadiomics and LIFEx, the only feature exhibiting excellent ICC is Volume (SHAPE), extracted from external resampled images. This may stem from the use of the same algorithm for image resampling, emphasizing the relevance of harmonizing the preprocessing steps. Considering texture features, we found that NGTDM features Busyness and Strength showed good ICC for both software, with internal and external resampling. In addition to the aforementioned features, texture features with good ICC for the internal and external resampling are RLNU (GLRLN) for LIFEx and ZP (GLZLM) for PyRadiomics. CT-MRI features with good or excellent ICC for PyRadiomics and LIFEx are shown in Table 4.

These results allow us to draw several considerations. As mentioned before, CT and MRI are two different techniques. Hence, our initial hypothesis was that most information embedded within the images would not be directly translatable from one image technique to the other. This hypothesis was corroborated by the analysis results. Vuong et al. had previously shown that SHAPE and texture features are highly correlated between PET/CT and PET/MRI [13]. Moving from nuclear medicine to diagnostic imaging, the same result was not yet known. Even though the correlation between certain CT and MRI SHAPE features could have been expected, as SHAPE features describe tumor morphological aspects, the correlation between CT and MRI texture features was not taken for granted. It is remarkable especially that two NGTDM features (Busyness and Strength) presented good ICC for both internal and external resampling and this was consistent across both radiomic software.

This study has limitations. First, the correlation we found is limited to the pathological process considered and the kind of images we compared, *i.e.*, NSCLC and contrast-enhanced CT and T1-weighted MRI. Second, while a diversity of CT equipment was used, only one 1.5-T MRI unit with a specific pulse sequence was employed. Third, this is a single-center study with a relatively small sample size. Nevertheless, it is a reliable proof of concept as first of all, currently, just a few centers have incorporated lung MRI into clinical practice, and secondly, it is relatively uncommon to have not only CT but also MRI acquisition for each patient.

In conclusion, we investigated the agreement between LIFEx and PyRadiomics software for two different imaging techniques, explored the correlation between CT-MRI corresponding features calculated with such software, and assessed how these relationships are affected by the resampling method. PyRadiomics and LIFEx are highly in agreement with each other for both MRI and CT (on average 90% of features showed ICC ≥ 0.75) and approximately 10% of MRI-CT related features (shape and texture) obtained from resampled images exhibited ICC ≥ 0.75. The impact of the resampling method was clear in the previous points. For both imaging modalities, we observed a decrease in agreement between the two radiomic tools when using their internal resampling methods.

To validate our results, further investigations using a wider multi-center cohort are necessary. These additional studies are also needed to confirm the “identity” of stable features in cross-modality and their generalizability as useful biomarkers. Furthermore, it is essential to single out sequences that are most suitable for radiomics with the aim of establishing a standardized acquisition protocol, especially for MRI images. A wider exploration of the potential of MRI radiomics in lung cancer patients through the use of other sequences (*e.g.*, unenhanced T1-weighted, T2-weighted, short-τ, diffusion metrics) could allow strengthening and/or expanding the identification of the most relevant features. Lastly, it would be interesting to evaluate the stability of our results across different segmentation methods.

As a final remark, in both MRI and CT cases, the study of physical principles and biomedical mechanisms underlying the radiomic features definition (a very challenging issue) and the extension of the presented approach to more modalities, including PET technique [14, 40], will possibly provide more robust biomarkers within a broader multimodality approach.

## Data Availability

The datasets generated and/or analyzed during the current study are not publicly available due to privacy restrictions but are available from the corresponding author on reasonable request.

## Abbreviations

CT: Computed tomography
GLCM: Gray level co-occurrence matrix
GLRLM: Gray-level run length matrix
GLZLM: Gray-level size zone matrix
IBSI: Image Biomarker Standardisation Initiative
ICC: Intraclass correlation coefficient
MRI: Magnetic resonance imaging
NGTDM: Neighboring gray-tone difference matrix
NSCLC: Non-small cell lung cancer
PET: Positron emission tomography
